# Determinants of pregnant women’s satisfaction with interactions with health providers at antenatal consultation in primary health care in Southern Mozambique in 2021: a cross-sectional study

**DOI:** 10.1186/s12884-024-06346-w

**Published:** 2024-02-26

**Authors:** Janeth Dulá, Sérgio Chicumbe, Maria do Rosário O. Martins

**Affiliations:** 1 Health Systems Program, Instituto Nacional de Saúde, estrada nacional n 0 1, vila de Marracuene, parcela 3943 CEP 0205-02, Marracuene district, Maputo Province, Mozambique; 2 Global Health and Tropical Medicine (GHTM), Institute of Hygiene and Tropical Medicine, Universidade Nova de Lisboa, Lisbon, 1349-008 Portugal

**Keywords:** Determinants, Satisfaction, Antenatal consultations, Primary Health Care, Mozambique

## Abstract

**Background:**

The Ministry of Health of Mozambique (MISAU) and the World Health Organization (WHO) recommend enhancing pregnant women’s satisfaction with health care services in order to advance maternal and child health. This study aims to assess the levels and determinants of pregnant women’s satisfaction regarding their interactions with antenatal care (ANC) providers, the services of which were provided at the primary health care level in southern Mozambique.

**Methodology:**

We conducted an observational, quantitative, and cross-sectional study from November 4 to December 10, 2021. A structured questionnaire was administered to pregnant women who attended ANC during that period. The characteristics of the participants were illustrated using descriptive statistics; to analyse pregnant women’s satisfaction determinants, we estimated crude and adjusted odds ratios (AOR) and 95% confidence intervals (95% CI) using logistic regression models. All analyses were performed in SPSS version 24 using a 5% significance level.

**Results:**

We selected 951 pregnant women with a mean age of 25 years old; 14% attained a secondary educational level, 36% were married or living in a marital relationship, and 85.9% reported being satisfied with their current ANC. Factors that reduced the odds of being satisfied were the following: an “insufficient” ANC duration (AOR = 0.173; 95% CI: 0.079, 0.381); inadequate ANC waiting area (AOR = 0.479; 95% CI: 0.265, 0.863); women’s perception about the existing norm of nonattendance in case of late arrival to the ANC (AOR = 0.528; 95% CI 0.292, 0.954); the perception of the existing norm that women are obliged to give birth in same health facility where ANC occurred (AOR = 0.481; 95% CI: 0.273, 0.846); and the perception that delivered ANC is not important for foetal health (AOR = 0.030; 95% CI:0.014, 0.066).

**Conclusions:**

Most of the pregnant women mentioned being satisfied with the ANC they received. The perception of short consultation duration, inadequate waiting spaces, strict linkage rules to specific health facilities and ANC norms, the perception that the received ANC is not relevant for foetal well-being are determinants of not being satisfied with ANC, and these determinants can be addressed by reorganizing ANC and, indeed, are modifiable by the improved paced implementation of the MISAU strategies for quality maternal and child health care.

**Supplementary Information:**

The online version contains supplementary material available at 10.1186/s12884-024-06346-w.

## Background

Antenatal care (ANC) remains one of the safest maternal care interventions aimed at significantly reducing maternal and perinatal morbidities [1]. It is routine health care provided to pregnant women by qualified health professionals, between conception and the onset of labour [1–5].

Antenatal care represents a fundamental role and an opportunity to offer care for the prevention and early detection of both maternal and foetal pathologies, allowing for the healthy development of the baby and reduction of the risks pregnant woman [1]. The WHO recommends that health services provide pregnant women with individualized, respectful, and pregnant woman-centred care in each contact [1].

The WHO *“Technical Consultation on Postpartum and Postnatal Care”* [6] estimates that worldwide, approximately 500,000 women die yearly due to complications of pregnancy and childbirth, and only 1% of these deaths occur in developed countries [5–8]. Maternal and neonatal deaths occur mostly in the period between delivery and the 7th day of life of the newborn. ‘’In most African countries, despite substantial investments and progress in improving the health of women and children, maternal and child mortality is still high despite substantial investments. Maternal and newborn outcomes in Africa can be further improved by delivering quality antenatal, peripartum, and postpartum health services” [9].

In this era of sustainable development goals, the Mozambican government’s priorities follow international priorities on maternal and child health [1, 7, 10]. Mozambique is a country with persistently high fertility rates, with approximately 5 children per woman in general, especially among those with low education, low socioeconomic status, and those living in rural areas [11, 12]. According to the most recent Demographic Health Survey (DHS) from 2023, in Mozambique, the use of health services is one of the main strategies to improve maternal and child health indicators, with the coverage of pregnant women having 4 or more antenatal consultations decreasing in the last few years, from 53% in the 2003 DHS to 49% in the 2022–23 DHS, and only 23% of them attended the first ANC before the end of the 1st trimester of pregnancy [9, 11, 12].

In 2017, Mozambique had 28 million inhabitants, 66.6% of whom lived in rural areas, and an annual growth of 2.9% was projected, according to census data [13]. Very low levels of education are prevalent in Mozambique’s population, with only 17.1% of women aged 15–49 attaining secondary education and 46.1% of the population living below the poverty line [9]. Although Mozambique had recorded considerable macroeconomic growth in previous years, the main economic activity in which the economically active population was engaged was subsistence agriculture; subsistence fishing is also practised in coastal areas (12.9% of the Mozambican population) [13].

Similary to several other low-income countries, in Mozambique, the majority of maternal deaths occur as a consequence of direct complications of pregnancy and childbirth (75%), with pregnancy and childbirth being the most critical periods for survival, both for women and newborns [10, 14]. Antenatal consultation are a key opportunity for contact between the “woman-foetus” dyad and health services, being one of the strategic components of long-term care for maternal, perinatal and unborn child health, with effects throughout the peripartum and postpartum health of mothers and newborns [1, 9]. ANC consultations are implemented as part of primary health care, and in Mozambique, these are typically provided by maternal and child health nurses [1, 9, 10].

Despite progress recorded in maternal and child health, Mozambique maintains uneven development and inequities in access to primary maternal and child health care [15]. In 2011, the ratio of maternal deaths in Mozambique was 458/100,000 live births [5], and after 5 years, it remained similar according to data from the 2017 population census [13], making it one of the highest in the world [16].

Antenatal health care and consultation allow for the identification of pregnancy risk factors [1]. Data from MISAU show that only 39.6% of women who had antenatal consultations in Mozambique were informed by health professionals about the signs of possible complications during pregnancy [17]. Since antenatal consultations are the first contact with the health system throughout pregnancy [1], any negative impression about quality and cost, including the opportunity cost of users waiting long hours to be attended to, may cause disengagement of pregnant women from health services. This disengagement may have sustained negative effects on the needed follow-up in other maternal and child health care periods, such as postpartum, neonatal, and vaccination. This disengagement from maternal and child health care services may also influence women’s decision to give birth in a health facility, thus increasing the risk of childbirth-related complications from the homebirths. Several other factors of satisfaction with health services are known, and they need to be contextually studied since there are still limited recent and comprehensive publications from sub-Saharan Africa about pregnant women’s satisfaction with antenatal consultations [7, 18–25].

The scarcity of specific and current research on Mozambican pregnant women’s satisfaction with ANC is a limitation to the processes of optimization of current health policies in Mozambique, more specifically the Mozambique health strategy – PESS 2014–2025 [10]. A lack of evidence about the levels and factors associated with pregnant women’s satisfaction with ANC is also prevalent in African countries in general. Understanding levels and factors of satisfaction with antenatal consultations is essential for the continuous improvement of the delivery of health services to pregnant women, and to inform about the need for timely adjustments in national health policies. This study’s usefulness lies in clarifying patterns, relationships, and the relative importance of satisfaction with ANC within the framework ofobjectives 1, 2 and 4 presented in PESS 2014–2025 [10].

## Methodology

### Study objectives

The aim of this study was to estimate the levels of pregnant women’s satisfaction with their interactions with ANC health providers, and to analyse its determinants in the southern region of Mozambique.

### Study design

This is an observational, quantitative, and cross-sectional study.

### Conceptual framework

The logical framework of this study refers to the Donabedian model [26], in which three domains of factors related to patient satisfaction are considered: [1] organizational components, [2] technical quality, and [3] interpersonal factors. These dimensions of user satisfaction with health services were considered the most relevant in some studies conducted in sub-Saharan Africa [27, 28]. Using the model, variables strongly associated with the satisfaction of ANC users were identified and included in the research: [1] sociodemographic and economic characteristics of the user; [2] organizational and operational aspects of ANC; [3] technical quality of the care received; and [4] communication and counselling factors.

### Setting

#### Study area and period

The study was implemented in 4 provinces of southern Mozambique: Maputo City, Maputo Province, Gaza and Inhambane. According to the 2018 National Infrastructure, Equipment, Human Resources, and Health Services Inventory (SARA 2018) [29], these 4 provinces had 391 primary care health units that provide antenatal consultation. The data were collected from November 4 to December 10, 2021.

#### Population

The target population was pregnant women who had attended up to two antenatal consultations in primary health centres in southern Mozambique. We consider that analysing the first and second consultations is a unique opportunity to determine how satisfaction may or may not guarantee the continued provision of services essential to the health of the mother and foetus.

#### Sample size and sampling methods

The sampling procedure was defined in two phases: In the first stage, we purposely selected 80 primary health facilities (HFs), corresponding to 20% of the total number of HFs that perform antenatal consultations in southern Mozambique, under the criterion of being HFs with the highest number of ANC users, according to data from the Mozambique Health Information System (SIS-MA).

Then, we computed the total sample size using the usual formula for proportions with *p* = 50% (which maximizes the sample size when the proportion of being satisfied is not known), a 95% confidence level, and a 5% margin error. We used the total population of 848,916 pregnant women who attended consultations in 2020 in the southern region of Mozambique according to SIS-MA data; we also considered a study design effect with a factor of 1.5, resulting in a minimum sample of 576 pregnant women [30].

### Participants

During 2 weeks of data collection (in phases; group 1: province and city of Maputo and group 2: Gaza and Inhambane), pregnant women who had attended up to 2 ANC visits and who consented to participate regardless of gestational age; the recruitment of participants was based on convenience. We included all participants who consented to participate in the interview, and all participants were interviewed immediately following their consultation. Each pregnant woman was interviewed individually, isolated from other patients and providers to minimize external effects on their answers. Prior to the interview, the pregnant women were informed about the purpose of the study, confidentiality, their rights and freedom to decide not to participate, and the nonexistence of any incentives for participation, and all of the women approached consented to participate in the study.

### Variables

#### Dependent variable

The dependent variable, the pregnant women’s satisfaction with their interaction with health providers at antenatal consultations, is defined as being equal to one if the pregnant woman is satisfied and zero if she is not. This is a composite variable that has been computed using factor analysis based on the 7 following dimensions of user perceptions:

1. Health professional was respectful *(0 = no and 1 = yes).*
2. Health professional was friendly *(0 = no and 1 = yes).*
3. Health professional was able to communicate well *(0 = no and 1 = yes).*
4. Nurses provided the best possible care *(0 = no and 1 = yes).*
5. Patient felt somehow humiliated or disrespected by the health professional *(0 = yes and 1 = no).*
6. Nurses gave priority to clients who offered “bribery” *(0 = yes and 1 = no).*
7. Health professionals demonstrated knowledge and competence *(0 = no and 1 = yes).*

1. We calculated the first factor scores and then dichotomized these variables using the following criteria: having an adequate value for the Kaiser‒Meyer‒Olkin test (KMO = 0.708), providing good sample adequacy in relation to the degree of observation between the variables; [2] Bartlett’s test of sphericity with *p* < 0.001, indicating that the variables are significantly correlated with the existence of good conditions to advance with the factor analysis; and [3] files with factor loadings lower than 0.40 were excluded. These criteria were used to reaffirm the adequacy of the sample, validating the use of factor analysis in this study (Supplementary Material Table 1.1).

#### Explanatory variables

Explanatory variables are organized in the following dimensions: [1] social and demographic variables: mother’s age (years), education, marital status, occupation with income, province of residence; [2] perception about the management and operation of the HF: privacy, reasons for adhering to the HF, expectation of waiting time prior to the consultation, satisfaction with the waiting time before the consultation, sufficient duration time of the ANC, adequate waiting area, attended on the same day even if arriving late, perceptions about early hours of the day (morning) with better service than in the afternoon, delivery of ANC until 3:30 p.m., nonattendance at same ANC implies consequently poor care, attendance of the ANC in the same HF does not affect acceptance to partum in the HF; [3] variables related to exposure to essential information and counselling during ANC: number of previous pregnancies, estimated date of delivery, place of delivery, plan for delivery, counselled on feeding practices, counselled on signs of pregnancy complications, asked if women had any questions or doubts, and offered to participate in the information and education sessions at HF; [4] variables related to clinical procedures: weight, height, belly examination, uterine height, blood pressure, syphilis test, intake of ferrous salt and folic acid, tetanus vaccination; and [5] Dimension of Relevance of prenatal consultation (Attending antenatal consultations is a duty of every mother, if you do not attend the appointments, the baby may, have compromised health);

In inferential, bivariate and multivariate analyses, aggregate variables were used instead of specific answers to questions posed to the participants obtained from the analysis of main components, namely: Composed index: Information and Counselling at ANC; Composed Index: Clinical Observation at ANC; and Composed Index: Prophylaxis at ANC (Supplementary Material Table 1.1).

#### Data collection tool

The interviews were conducted by trained surveyors using a structured electronic questionnaire in the Kobo toolbox. The study’s data collection tool was adapted from questionnaires applied in similar studies carried out in Africa [18, 19, 22, 31].

### Data analysis

A descriptive analysis includes absolute and relative frequencies for the categorical variables (sociodemographics, management and operation characteristics, Composed index: Information and Counselling at ANC; Composed Index: Clinical Observation at ANC; and Composed Index: Prophylaxis at ANC, and pregnant women’ satisfaction). The respondent’s age was described using the mean, median, standard deviation, and quartile interval.

To analyse determinants of pregnant women’s satisfaction with their interaction with health providers at ANC, we estimated crude and adjusted odds ratios (AOR) and respective 95% confidence intervals (95% CI) using logistic regression models. We included in the multivariable model variables with a *p value* < 20% in the simple model [18, 19, 22, 31]. For statistical tests, we used a 5% significance level. All analyses were performed in the *Statistical Package for Social Science (*SPSS) version 24.

### Ethical considerations

The study conformed to and was implemented following national and international ethical standards, especially the Declaration of Helsinki, having been approved by the National Committee of Bioethics for Health of Mozambique, with reference 761/CNBS/2021. The participants informedly and freely consented to their inclusion in the study.

## Results

The 80 health facilities were distributed as follows: Inhambane Province [10], Gaza Province [31], Maputo Province [24], and Maputo City [14]. A total of 951 pregnant women agreed to be interviewed. The computation of the dependent variable suggested that 85.9% (*n* = 817) were satisfied with their interaction with ANC providers.

### Sociodemographic profile

The participants’ sociodemographic profile is described in Table 1. The majority (44%) of the pregnant women were from Gaza Province, and the mean age was 25 years (SD = 6); although 64% of the respondents spoke the official and schooling language-Portuguese, a significant proportion (36%) did not speak this language. The majority (86%) can read letters in Portuguese, and only 14% have an educational level above the 8th grade (secondary level).

**Table 1:** Sociodemographic Characteristics of respondents in public health centers, Southern Mozambique (*n* = 951)

		N	%
Province	Inhambane	135	14.2
	Gaza	419	44.1
	Maputo Province	256	26.9
	Maputo Downtown	141	14.8
	Total	951	100
THE INTERVIEW IS BEING CONDUCTED IN PORTUGUESE	Portuguese	604	63.5
	Xichangana	314	33.0
	Bintongo	4	0.4
	Xitswa	29	3.0
	Total	951	100
Age	Media	25	
	Median	24	
	DP	6	
	Range	31	
	Mininum	14	
	Maximum	45	
	Missing	4	
	Total	947	
Can you read and write a letter in Portuguese	No	131	13.8
	Yes	820	86.2
	Total	951	100
Educational level	< Grade 7	819	86.1
	Grade 8 +	132	13.9
	Total	951	100.0
Marital status	Currently Married	338	35.5
	Currently unmarried	613	64.5
	Total	951	100
Parity	Small parity	833	94.1
	Great parity	52	5.9
	Total	885	100.0
	Missing	66	6.9
	Total	951	100
Work that provides income	No	793	83.4
	Yes	158	16.6
	Total	951	100

More than one-third (36%) are currently married or living in a marital relationship, and 94% have small parity (up to 3 children); most pregnant women do not have a paid job (83%).

### Management and operation of health facilities

The majority (85%) of women reported privacy during ANC and an adequate waiting area for ANC (77%). Regarding the main reason for choosing to use one HF for ANC over another HF, most (81%) reported the HF’s proximity to their home, followed by the HF’s quality of care (11%). Regarding the waiting time, 58% reported a shorter waiting time for ANC than expected, and 19% reported dissatisfaction with the waiting time for ANC.

Additionally, 42% of the pregnant women mentioned that late arrival to consultation implied not being attended on the same day and 48% that they had a preference for visits in the morning rather than in the afternoon. Regarding the working hours at the HF, 63% of pregnant women reported that service was not provided in the afternoon, and a large proportion reported having had enough time (93%) with the nurse during the consultation. For noncompliance with the appointment date, 43% of the women reported verbal mistreatment if the appointment date was missed.

It was also found that 42% of study participants had perceptions that they might not be assisted for delivery in a particular HF if they did not attend ANC in the very same HF. Approximately 92% knew that attending ANC is a duty of every mother, and 93% reported being aware of the importance of the consultation for foetus health (Table 2).

**Table 2:** Waiting Time and Operability in public health centers, Southern Mozambique (*n* = 951)

		N	%
Privacy	No	144	15.1
	Yes	807	84.9
	Total	951	100.0
Reason for choosing this health facility instead of another	Close to Home	768	80.8
	Quality Service	100	10.5
	Other	83	8.7
	Total	951	100.0
You expected to spend more time or less time than you waited	Equal	125	13.1
	Longer	273	28.7
	Less time	553	58.1
	Total	951	100.0
Satisfied with the waiting time	No	178	18.7
	Yes	773	81.3
	Total	951	100.0
Sufficient time at ANC	No	71	7.5
	Yes	880	92.5
	Total	951	100.0
Suitable waiting area	No	216	22.7
	Yes	735	77.3
	Total	951	100.0
When arriving late, the patient is seen that day	No	400	42.1
	Yes	551	57.9
	Total	951	100.0
In the morning, the care is better for patients than in the afternoon	No	491	51.6
	Yes	460	48.4
	Total	951	100.0
Nurses provided antenatal consultations until 3:30 p.m.	No	602	63.3
	Yes	349	36.7
	Total	951	100.0
If you do not attend ANC may be badly assisted	No	546	57.4
	Yes	405	42.6
	Total	951	100.0
If you do not attend antenatal consultations in this HF, you can be assisted for delivery here	No	281	42.3
	Yes	384	57.7
	Total	665	100.0
	Missing	2	286
	Total	951	
Attending antenatal consultations is a duty of every mother	No	73	7.7
	Yes	878	92.3
	Total	951	100.0
If you do not attend the appointments, the baby may have compromised health	No	63	6.6
	Yes	888	93.4
	Total	951	100.0

### Information and counselling

During consultation, less than half (44%) reported being asked about previous pregnancies. Only 33% were informed about their estimated date of delivery, and most respondents (88%) reported having been told about where to give birth. 71% did not receive counselling on the plan for childbirth, 73% did not receive antenatal baby feeding counselling, and the majority reported not having attended the mass information and education sessions (80%) on the day of interview. Concerning pregnancy complication counselling, 88% of the pregnant women reported not having received counselling or information, and only 26% were asked if they had any questions to raise during ANC. The information and counselling received in the ANC was ranked in quintiles for further analysis, and 41% of women respondents were in the lowest quintile (Table 3).

**Table 3:** Information and Counsilling in antenatal care in public health centers, Southern Mozambique (*n* = 951)

		N	%
Previous pregnancy	No	537	56.5
	Yes	414	43.5
	Total	951	100.0
Estimated date of delivery	No	639	67.2
	Yes	312	32.8
	Total	951	100.0
Information on place of delivery	No	835	87.8
	Yes	116	12.2
	Total	951	100.0
Plan for childbirth	No	677	71.2
	Yes	274	28.8
	Total	951	100.0
Counselling on food	No	688	72.6
	Yes	260	27.4
	Total	948	100.0
	Missing	3	0.32
	Total	951	100
Counselling about signs of complications	No	832	87.5
	Yes	119	12.5
	Total	951	100.0
Asked if she had doubts	No	703	73.9
	Yes	248	26.1
	Total	951	100.0
Attended the morning lecture	No	762	80.1
	Yes	189	19.9
	Total	951	100.0
Quintile Information and Counselling at ANC	Lowest	233	24.5
	Low	153	16.1
	Middle	181	19.0
	High	192	20.2
	Highest	192	20.2
	Total	951	100.0

### Medical procedures (screening and essential prophylaxis)

The results show that 95% of the pregnant women had their weight assessed and half (53%) had their height measured on the day of interview. On the other hand, 93% reported having their belly examined, 87% had their uterine height measured, and 80% had their blood pressure taken. Among those who were tested for syphilis on the day of the interview (41.3%), 91% were counselled on what to do depending on the result of the test. 81% received ferrous salt and folic acid tablets during the antenatal visit; medical procedures reported as performed during ANC were ranked in quintiles for further analysis, and 40% of pregnant women were in the lowest and lowest quintiles (Table 4).

**Table 4:** Clinical Screening in antenatal care in public health centers, Southern Mozambique (*n* = 951)

		N	%
Measured Weight	No	52	5.5
	Yes	899	94.5
	Total	951	100.0
Measured Height	No	445	46.8
	Yes	506	53.2
	Total	951	100.0
Examined the belly	No	71	7.5
	Yes	880	92.5
	Total	951	100.0
Measurement of Uterine Height	No	128	13.5
	Yes	823	86.5
	Total	951	100.0
Blood Pressure Measurement	No	187	19.7
	Yes	764	80.3
	Total	951	100.0
Performed Syphilis test	No	542	58.7
	Yes	381	41.3
	Total	923	100.0
	Missing	28	2.9
	Total	951	100.0
Provided the result and explanation of the Syphilis test	No	35	9.3
	Yes	342	90.7
	Total	377	100.0
	(Not tested)	574	60.4
	Total	951	100.0
Received ferrous salt with folic acid	No	185	19.6
	Yes	760	80.4
	Total	945	100.0
	Missing		0.6
	Total		100.0
Quintile Clinical Observation at ANC	Lowest	183	20.0
	Low	185	20.2
	Middle	170	18.5
	High	180	19.6
	Highest	199	21.7
	Total	917	100.0
	Missing	34	3.6
	Total	951	100.0
Quintile Prophylaxisxia at ANC	Lowest	179	19.5
	Low	194	21.2
	Middle	176	19.2
	High	185	20.2
	Highest	183	20.0
	Total	917	100.0
	Missing	34	3.6
	Total	951	100.0

### Interaction between health providers and clients

Most respondents (95.7%) considered that the nurses did their best on providing ANC; however, 14.2% reported priority attendance after “bribing” the nurse. 94% of the pregnant women deemed the nurses competent, and most felt health professionals were respectful (94.7%); nurses were empathetic (91.9%), considered the health professional’s communication adequate (95.4%), and 2.7% felt humiliated by the health provider (Table 5).

**Table 5:** Satisfaction with antenatal care in public health centers, Southern Mozambique (*n* = 951)

		N	%
Nurses provide the best care possible	No	41	4.3
	Yes	910	95.7
Nurses give priority to clients who give “bribery”	No	816	85.8
	Yes	135	14.2
	Total	951	100
Health professional demonstrates knowledge and competence	No	60	6.3
	Yes	888	93.7
	Total	948	100
	Missing	3	0.3
	Total	951	100
Health professional shows respect	No	50	5.3
	Yes	901	94.7
	Total	951	100
Health professional shows sympathy	No	77	8.1
	Yes	874	91.9
	Total	951	100
Felt somehow humiliated or disrespected by the health professional	No	925	97.3
	Yes	26	2.7
	Total	951	100
Health professional was able to communicate well	No	44	4.6
	Yes	907	95.4
	Total	951	100.0
Satisfaction with ANC	No	134	14.1
	Yes	817	85.9
	Total	951	100

### Determinants of satisfaction with interaction with health providers at ANC

Results from logistic regression estimations, crude, and adjusted odds ratios and respective 95% CIs are shown in Table 6. The variables included in the multivariate analysis are those that, in the bivariate analysis, were associated *(p = < 0.05*) with the dependent variable “satisfaction”. These are: Province, Sufficient time at ANC; Suitable waiting area; when arriving late the patient is seen that day; Nurses provide antenatal consultations until 3:30 p.m.; If you do not attend antenatal consultations in this HF, you can be assisted for delivery here; and If you do not attend the appointments, the baby may have compromised health.

**Table 6:** Women’s characteristics and its association with antennal consultation in the Southern Region of Mozambique (*n* = 951)

		*p*	OR	COR (95% CI)		*p*	AOR	AOR (95% CI)	
				Min	Max			Min	Max
Province*	Inhambane vs. Maputo City	0.102	1.659	0.904	3.044	0.246	1.848	0.655	5.219
	Gaza vs. Maputo City	0.000	2.478	1.509	4.068	0.663	1.199	0.531	2.707
	Maputo Province vs. Maputo City	0.011	1.995	1.173	3.394	0.238	1,684	0.709	3.999
Age	Age vs. increase	0.400	0.987	0.958	1.017	-			
Can you read and write a letter in Portuguese	Can read and write vs. doesn’t know	0.351	1.313	0.741	2.326	-			
Educational level	< Grade 7 vs. Grade 8 +	0.333	0.754	0.426	1.336	-			
Marital status	currently married vs. currently unmarried	0.789	0.949	0.650	1.388	-			
Parity	Small parity vs. Great parity	0.846	0.926	0.425	2.015	-			
Work that provides you with income	With work that provides you with income vs. No work	0.493	1.180	0.735	1.892	-			
Privacy	It is not the only patient to be seen vs. the only patient	0.222	0.743	0.462	1.197	-			
Reason for choosing this health facility instead of another	reason: Quality Service vs. other reasons	0.420	0.743	0.361	1.529	-			
	reason: Close to Home vs. other reasons	0.238	0.594	0.250	1.413				
Satisfied with the waiting time	Dissatisfied with the waiting time vs. Satisfied	0.350	0.808	0.516	1.264	-			
Attendance at antenatal consultations	It’s not hard to attend ANC vs. it’s hard	0.285	0.625	0.264	1.480	-			
Sufficient time at ANC*	Didn’t spend enough time with the nurse vs. Spent enough time	**0.000**	0.149	0.090	0.249	**0.000**	0.173	0.079	0.381
Suitable waiting area*	Not suitable vs. is suitable	**0.000**	0.464	0.313	0.686	**0.014**	0.479	0.265	0.863
When arriving late, the patient is seen that day*	Not attended that day vs. is attended	**0.002**	0.558	0.386	0.805	**0.034**	0.528	0.292	0.954
In the morning, nurses serve patients better than in the afternoon	Do not attend better patient’s vs. attends better	0.600	0.907	0.629	1.308	-			
Nurses provide antenatal consultations until 3:30 p.m.*	Do not provide until 3:30 p.m. vs. Provide	**0.001**	0.472	0.309	0.723	0.177	0.642	0.337	1.222
If you do not attend ANC may be badly assisted	Don’t Speak Badly vs. Speak	0.697	0.929	0.641	1.346	-			
If you do not attend antenatal consultations in this HF, you can be assisted for delivery here*	You can’t give birth here vs. you can give birth	**0.044**	0.627	0.398	0.988	**0.011**	0.481	0.273	0.846
Attending antenatal consultations is a duty of every mother	It’s not every mother’s duty VS it’s a duty	0.549	0.820	0.429	1.568	-			
If you do not attend the appointments, the baby may have compromised health*	the baby can’t have compromised health vs. the baby can	**0.000**	0.037	0.020	0.068	**0.000**	0.030	0.014	0.066
Quintile Information and Counselling at ANC	Lowest vs. Highest	0.639	1.147	0.647	2.031	-			
	Low vs. Highest	0.174	0.670	0.376	1.194				
	Medium vs. Higher	0.704	0.893	0.499	1.598				
	High vs. Highest	0.762	1.096	0.605	1.987				
Quintile Clinical Observation at ANC	Lowest vs. Highest	0.960	0.986	0.559	1.738	-			
	Low vs. Highest	0.541	1.202	0.667	2.163				
	Medium vs. Higher	0.343	0.765	0.440	1.331				
	High vs. Highest	0.129	1.636	0.866	3.090				
Quintile Prophylaxis at ANC	Lowest vs. Highest	0.512	1.216	0.678	2.183	-			
	Low vs. Highest	0.502	1.217	0.686	2.157				
	Medium vs. Highest	0.365	1.318	0.725	2.396				
	High vs. Highest	0.630	1.152	0.649	2.044				

*The variables that are included in the multivariate analysis are: Province; Sufficient time at ANC; Suitable waiting place; When arriving late, the patient is seen that day; Nurses provide antenatal consultations until 3:30 p.m.; If you do not attend antenatal consultations in this HF, you can be assisted for delivery here; If you do not attend the appointments, the baby may have compromised health

Abbreviations: ANC, antenatal care

As seen, pregnant women who were less likely to be satisfied with the interaction with ANC were those who considered the duration of the antenatal consultation to be insufficient (AOR = 0.173; 95% CI: 0.079, 0.381); those who found the waiting area inadequate (AOR = 0.479; 95% CI: 0.265, 0.863) and those who perceived that late arrivals implied not being attended to (AOR = 0.528; 95%CI: 0.292, 0.954).

Women who perceived going to a different HF for ANC implied not being allowed to deliver their baby in that HF (AOR = 0.481; 95%CI: 0.273, 0.846) were less likely to be satisfied with the interaction with ANC providers than women who considered being allowed to give birth in any HF even if they did not attend ANC in that HF. Participants unable to report the importance of ANC for foetal health were also less likely to be satisfied with their interaction with ANC providers (AOR = 0.030; 95%CI: 0.014, 0.066).

## Discussion

This is the first study to be published on the determinants of satisfaction of pregnant women with health providers at antenatal consultations in Mozambique. Antenatal care is a key health service for women’s adherence and retention in the national health care service [1, 10]. Antenatal consultations provide health promotion, prevention, screening, treatment and other important prophylaxis for the health of the mother and the foetus [32]. Quality ANC requires satisfaction with the consultation, so satisfaction with ANC is an important determinant of the entire pregnancy outcome, including safety in childbirth, access to essential obstetric care, and later, family planning, which together can contribute to the reduction of maternal mortality [1, 33].

We aimed specifically to study the levels and determinants of pregnant women’s satisfaction with their interactions with health providers at ANC consultations through users’ perceptions of the various dimensions of ANC. In this study, we included only women who had fewer than two antenatal visits, as we aimed to assess satisfaction with their first or second contact with ANC. This approach is different from several studies [24, 25] that considered it a limitation to measure satisfaction in clients who attended few consultations. However, ANC users’ experiences and perceptions with the first or initial consultation is crucial for attendance of follow-up or consecutive consultations; thus, analysing the first and second consultations is a unique opportunity to determine how satisfaction may or not secure continuous provision of essential services for the health of the mother and foetus.

This survey reports satisfaction with interactions with health providers of antenatal services amongst 85.9% of pregnant women in the southern region of Mozambique. These findings are similar to those reported by studies from Nigeria, Ethiopia, and South Africa [20–25] and suggest substantial improvements in pregnancy public health services.

Several studies [18, 20, 22, 27, 34] align with our findings, showing that the waiting areas for consultation and the duration time of consultation are determinants of satisfaction. Factors related to the waiting time and the operation of health services, —such as attendance for late arrivals and antenatal consultations over entire health facilities’ working hours until 3:30 p.m.; —impacted pregnant women’s satisfaction with ANC. Similar results were found in countries with health systems similar to that of Mozambique, where the degree of patient satisfaction with the health services provided in HF is highly related to the waiting time [19–21, 34, 35].

Similarly, a study analysing the relationship between the waiting time for consultation and satisfaction with the health services provided in the United States [36] concluded that there is an inverse and statistically significant association between long waiting times and patient satisfaction. Indeed, the waiting time can be seen as a cost to the patient to be attended by a health professional, and the greater this investment of time is, the lower the patient’s satisfaction with the health service. Considering not only the waiting time but also the duration of the consultation with the health professional, our study shows that the highest levels of dissatisfaction are associated with not only a long waiting time but also a short duration of the consultation, and both factors were previously described in the Mozambican context [34, 36].

Our study found that most pregnant women perceive that (i) nurses do not provide consultations until 3:30 p.m.; (ii) that when patients arrive late, they are not attended; and that (iii) if they do not attend antenatal consultation at a HF, they will not be assisted for childbirth at the same HF. These misperceptions regarding the operation and availability of services are determinants of satisfaction and consequently can prevent pregnant women’s flexibility in attendance at health services. It is worth mentioning that the national guidelines in Mozambique recommend that HF operate from 7:30 a.m. to 3:30 p.m., without interruptions throughout the day, and that antenatal consultations shall be organized as a “one-stop shop”, —that is, all services, including health promotion, screening, prophylaxis, and treatment, are performed in the same office. Therefore, nurses and services should be available throughout business hours to serve users [37].

Although the level of clinical screening at ANC did not impact satisfaction in the adjusted analysis, our study shows increased levels of clinical screening provision to pregnant women at the studied HF compared with lower levels reported by the Ministry of Health in 2009. However, accounting for results on information and counselling, our results suggest that there was no increase in counselling and information provision to pregnant women in the last 10 years [17]. Conversely, there was an increase in the provision of prophylaxis packages, screening and treatment, but the levels of service provision did not increase in dimensions such as communication, information, and education in health. Studies carried out in both developing and developed countries [20, 21, 27, 34, 38] show that information and counselling are important determinants of satisfaction with antenatal care.

Finally, although levels of clinical assessment or observation did not determine satisfaction with ANC in our adjusted analysis, clinical observation is considered a key procedure by women. Our study differs from a few others since clinical observations were a determinant of satisfaction with services in two studies conducted in Nigeria [23, 34].

### Limitations of the study

A possible limitation of our study was the bias in the selection of the study site; our study was conducted in health facility environment, which may have influenced interviewees to give socially desirable responses during the interview. Respondents tended to positively assess the behaviour of health professionals since the results showed that almost the entire of the study respondents were satisfied with them, but when asked about the services received, the waiting time, and information and counselling, there was a variation in the answers and a dissatisfaction with these specific components.

However, this bias was highly minimized because the interviews, apart from having been carried out in consultation offices, and at the end of the consultation, were conducted with guaranteed privacy; in addition, the interviewers were detached from health service provision since these were experienced collaborators of the National Institute of Health, and it was clear they were not part of the HF teams where the study was conducted. Additionally, satisfaction in this study was a composite indicator based on questions asked indirectly. The advantage of conducting interviews in the HF environment to the detriment of the community was that memory bias was minimized since the interviews were conducted at the end of antenatal consultations.

Finally, we included only public health facilities in the study setting. This may not be representative of pregnant women who frequent private facilities; however, the vast majority of pregnant women attended prominent public health facilities in Mozambique.

### Implications

The large and continuous investment in improving the availability and readiness of sexual and reproductive health services over the past 20 years, particularly antenatal and maternity services [10, 29, 39, 40], might have contributed to high levels of women’s satisfaction with ANC. However, long waiting times, lack of proper health education, information, and communication continue to be determinants of satisfaction, and a comparison of the previous two decades shows that there have been no improvements in these determinants [9–11, 15, 39]. It is therefore urgent and crucial that reforms and revisions to the current policy are made so that the quality and satisfaction of the services can be optimized. The identified factors of satisfaction with ANC depend on adjusted multisectoral policies. Most pregnant women in southern Mozambique are young, with low education and low socioeconomic status, so we recommend adapting Information, Education and Communication actions to the low level of literacy of women; It should also be ensured that audiovisual materials about pregnancy reach pregnant women outside of the health facility environment.

It is also recommended to implement strategies that ensure compliance and completion of appointments without cancellation, and for women with low economic status, that antenatal consultations are carried out in mobile brigades to reduce this burden. It is necessary to increase the consultation time and attend to women outside the morning hours, because these are the factors that are associated with satisfaction; Therefore, we propose the implementation of interventions to improve the management of the flow of patients and the increase of human resources.

And finally, a finding that we consider positive from the study is that the clinical procedures are routinely performed, so it is recommended to maintain the good clinical evaluation practices that are carried out in primary care health units. The results might be transferable countrywide as well as to other African countries with similar developmental and health organization contexts.

## Conclusions

Most pregnant women in southern Mozambique are satisfied with their interactions with health providers at antenatal consultations, but dissatisfaction arises from long waiting times and operational features of health facilities. A notable number lack sufficient information and counselling on several aspects of maternal health. Given the significance of initial antenatal visits, special attention during these sessions is crucial for encouraging return visits. Investing in training health professionals for effective communication is urgently recommended to enhance user satisfaction. Moreover, efforts are needed to align services with national antenatal care policies, contributing to Mozambique’s goal of increase the quality of care for pregnant women, and reducing maternal and infant mortality outlined in the sustainable development goals. Future research, including qualitative, experimental studies, and case‒control studies, is necessary to delve deeper into the impact of satisfaction on health services.

### Electronic supplementary material

Below is the link to the electronic supplementary material.

Supplementary Material 1

## Data Availability

The datasets used and/or analysed during the current study available from the corresponding author on reasonable request.

## Abbreviations

AOR: Adjusted Odds Ratio
ANC: Antenatal Care
HF: Health facilities
KMO Test: Kaiser-Meyer-Olkin Test
MISAU: Ministry of Health of Mozambique
95% CI: 95% confidence intervals
PESS: Mozambique Health Sector Strategy Plan–2014–2019 [2025]
WHO: World Health Organization
OR: Odds Ratio
SARA: National Infrastructure, Equipment, Human Resources, and Health Services Inventory
SIS-MA: Data from the Mozambique Health Information System
