# Supplementary material for: Determinants of pregnant women’s satisfaction with interactions with health providers at antenatal consultation in primary health care in Southern Mozambique in 2021: a cross-sectional study
Source: BMC Pregnancy Childbirth. 2024 Feb 26;24:165. doi: 10.1186/s12884-024-06346-w (PMC10895729; doi:10.1186/s12884-024-06346-w)
Supplement: Supplementary file 1 — Supplementary Material 1 [file 12884_2024_6346_MOESM1_ESM.docx]

Factor Analysis

1. To measure the satisfaction of pregnant women with ANC, exploratory factor analysis was used, starting from items on essential information and counselling at ANC (6 ITEMS), on essential clinical screening at ANC (8 ITEMS) and on satisfaction with ANC (7 ITEMS).
2. For the exploratory factor analysis on Satisfaction with ANC (7 ITEMS), it was possible to find that the Kaiser‒Meyer‒Olkin test (KMO= 0.708) is good, and the Bartlett sphericity test has a *p value < 0.001*, indicating that the variables are significantly correlated with the existence of good conditions to advance with the factor analysis. First, the commonality test was performed, where five items with factor loadings lower than 0 were initially removed, 40, namely, nurses are doing their best to serve patients, nurses give priority to patients who give bribery, perception about knowledge and competence of the professional who assisted you, any professional treated you in a humiliating or disrespectful way, and perception about communication skill of the health professional who assisted you. Where it was possible to verify in the matrix of factors 1 dimension (factor), namely: (1) perception about respect of the professional during the antenatal consultation/perception about the sympathy of the health professional/perception about communication of the health professional who assisted you in this antenatal consultation. The total variation explained by the 7 items grouped in one factor was 28% (table 3.1).

|  |  |  |
| --- | --- | --- |
| Table 1.1. Factor Analysis of Satisfaction | |  |
| **Satisfaction Factors** | Factor |  |
|  | 1 | Comunalidade |
| How would you classify the sympathy of the health professional | **0.759** |  |
| How would you classify the respect of the professional during the antenatal consultation | **0.819** |  |
| How would you rate the communication skill of the health professional who assisted you in this antenatal consultation | **0.749** |  |
| Are nurses doing their best to serve patients | **0.379** |  |
| At some point today in the health facility some professional treated you in some way that you felt humiliated or disrespected |  |  |
| Do nurses give priority to patients who give bribery |  |  |
| How would you rate the knowledge and competence of the professional who assisted you |  |  |
| Extraction Method: Principal Axis Factoring. | |  |
| a. Attempted to extract 3 factors. More than 25 iterations required. (Convergence=.001). Extraction was terminated. | |  |
| Kaiser-Meyer-Olkin Measure of Sampling Adequacy. | | 0.708 |
| Bartlett's Test of Sphericity | Approx. Chi-Square | 698.552 |
|  | df | 21 |
|  | Itself. | 0.000 |
|  |  |  |

3. For the exploratory factor analysis on essential information and counselling at ANC (6 ITEMS), it was possible to find that the Kaiser‒Meyer‒Olkin test (KMO= 0.638) is good, and the Bartlett sphericity test has a *p value < 0.001*, indicating that the variables are significantly correlated with the existence of good conditions to advance with the factor analysis. First, the commonality test was performed where four items with factorial loads less than 0.40, namely, health professional informed you of the estimated date for delivery, the health provider talked about a plan for childbirth, health professional has asked if you had doubts and have attended the morning lecture. Where it was possible to verify in the matrix of factors 2 dimensions (factors), namely: (1) health professional told you about signs of complications/the health provider talked about a plan for childbirth/the health professional informed you the estimated date for delivery; (2) During today's consultation, were you asked about any previous pregnancies: such as the number of pregnancies, the results and possible complications. (table 3.2).

Table 1.2. Factor Analysis of Information and Counselling

| **Information and Counselling Factors** | Factor | |  |
| --- | --- | --- | --- |
|  | 1 | 2 | Commonality |
| The health professional told you about SIGNS OF COMPLICATIONS in pregnancy | **0.680** |  |  |
| The health professional talked about a PLAN FOR CHILDBIRTH | **0.474** |  |  |
| The health professional informed you of the ESTIMATED DATE OF DELIVERY | **0.413** |  |  |
| Did the health professional ask you if you had any doubts? |  |  |  |
| The health professional asked you about any PREVIOUS PREGNANCY | 0.515 | **0.520** |  |
| Did you attend the lecture this morning |  |  |  |
| Extraction Method: Principal Axis Factoring. | | |  |
| a. Attempted to extract 2 factors. More than 25 iterations required. (Convergence=.006). Extraction was terminated. | | |  |
| Kaiser-Meyer-Olkin Measure of Sampling Adequacy. | | 0.638 |  |
| Bartlett's Test of Sphericity | Approx. Chi-Square | 357.254 |  |
|  | df | 15 |  |
|  | Itself. | 0.000 |  |

4. For the exploratory factor analysis of Clinical Screening with ANC (8 ITEMS), it was possible to find that the Kaiser‒Meyer‒Olkin test (KMO= 0.718) is good, and the Bartlett sphericity test has a *p value < 0.001*, indicating that the variables are significantly correlated with the existence of good conditions to advance with the factor analysis. First, the commonality test was performed. First, the communality test was carried out with six items with factor loadings lower than 0.40, and it was possible to verify 2 dimensions (factors) in the factor matrix, namely: (1) Did the health professional examine your belly/During the consultation, did they measure your WEIGHT/Did the health professional measure the uterine height and/During the consultation, did they measure you blood pressure? (2) Did you receive tetanus vaccine and/During the consultation, did the health professional perform a syphilis test? (table 3.3).

Table 1.3. Factor Analysis of Clinical Screening

| **Clinical Screening Factors** | Factor | |
| --- | --- | --- |
|  | 1 | 2 |
| Did the health professional EXAMINE YOUR BELLY | **0.775** |  |
| During the consultation, did they measure your WEIGHT | **0.653** |  |
| Did the health professional measure the UTERINE HEIGHT | **0.589** |  |
| During the consultation, did they measure you BLOOD PRESSURE | **0.438** |  |
| During the consultation, did they measure your HEIGHT |  |  |
| Did you receive tetanus vaccine |  | **0.587** |
| During the consultation, did the health professional perform a syphilis test |  | **0.474** |
| During today's appointment, did the professional hand you tablet (or a prescription) of ferrous salt with folic acid? |  |  |
| Extraction Method: Principal Axis Factoring. | | |
| a. Attempted to extract 1 factor. More than 25 iterations required. (Convergence=.002). Extraction was terminated. | | |
| Kaiser-Meyer-Olkin Measure of Sampling Adequacy. | | 0.718 |
| Bartlett's Test of Sphericity | Approx. Chi-Square | 1017.130 |
|  | df | 28 |
|  | Itself. | 0.000 |
